# Lung Multidisciplinary Team Decision-Making Challenges in Perioperative NSCLC: Borderline Resectability and Discordant Responses Through the Lens of Real-World Scenarios

**DOI:** 10.3390/cancers18203377

**Published:** 2026-10-19

**Authors:** Diego Luigi Cortinovis, Stefania Canova, Maria Ida Abbate, Francesca Colonese, Luca Sala, Alice Ilari, Sara Mambrito, Piergiorgio Solli, Giovanni Leuzzi, Fabrizio Luppi, Stefano Arcangeli, Francesca Bono, Cammillo Roberto Talei Franzesi, Luca Guerra, Francesco Petrella

**Affiliations:** 1 Medical Oncology, Fondazione IRCCS San Gerardo dei Tintori, 20900 Monza, Italyalice.ilari@irccs-sangerardo.it (A.I.);; 2 School of Medicine and Surgery, University of Milano-Bicocca, 20126 Milan, Italy; 3 Division of Thoracic Surgery, Fondazione IRCCS Istituto Nazionale dei Tumori, 20133 Milan, Italy; 4 Pneumology, Fondazione IRCCS San Gerardo dei Tintori, 20900 Monza, Italy; 5 Radiation Oncology, Fondazione IRCCS San Gerardo dei Tintori, 20900 Monza, Italy; 6 Pathology Unit, Fondazione IRCCS San Gerardo dei Tintori, 20900 Monza, Italy; 7 Department of Radiology, Fondazione IRCCS San Gerardo dei Tintori, 20900 Monza, Italy; 8 Department of Nuclear Medicine, Fondazione IRCCS San Gerardo dei Tintori, 20900 Monza, Italy; 9 Thoracic Surgery, Fondazione IRCCS San Gerardo dei Tintori, 20900 Monza, Italy

**Keywords:** perioperative setting, chemo-immunotherapy, locally advanced disease, multidisciplinary team, surgical operability, stage II–III NSCLC

## Abstract

Background: Resectable or potentially resectable stage II–III NSCLC encompasses heterogeneous clinical scenarios. The introduction of neoadjuvant and perioperative strategies has expanded treatment options while increasing the complexity of multidisciplinary decision-making and the need to reassess treatment pathways over time. Methods: We conducted a single-centre retrospective descriptive analysis of 101 patients with clinical or pathological stage II–III NSCLC discussed within our thoracic multidisciplinary team (MDT) between 2023 and 2025. Using MDT meeting records supplemented by medical records, we analysed decision-making trajectories and the main reasons for changes in treatment strategy. Results: Three major decision points emerged: selection of preoperative treatment, reassessment of resectability and operability after preoperative treatment, and postoperative assessment of recurrence risk and feasibility of adjuvant treatment. Treatment pathways were modified according to disease response or progression, surgical feasibility, comorbidities, treatment-related toxicity, pathological findings, and postoperative recovery. Particularly challenging scenarios included heterogeneous stage II disease, borderline resectability, and discordant responses after induction therapy. Conclusions: Perioperative management of stage II–III NSCLC requires dynamic, longitudinal multidisciplinary decision-making rather than a single treatment decision at diagnosis. Integrated reassessment of clinical, pathological, and molecular disease characteristics, treatment response, patient fitness, and surgical feasibility may help individualise treatment pathways.

## Simple Summary

The management of stage II–III non-small cell lung cancer has become increasingly complex with the introduction of preoperative and perioperative systemic treatments, molecular testing, and more individualized surgical strategies. This study aims to understand how treatment plans are actually selected and adapted in routine multidisciplinary practice, focusing on the main points at which the initial strategy changes. We retrospectively analyzed 101 consecutive patients with stage II–III disease discussed by a thoracic multidisciplinary team between 2023 and 2025. The analysis identified three major decision points: selection of preoperative treatment, reassessment of eligibility for surgery, and feasibility of postoperative treatment. The findings show that treatment pathways are often modified because of disease response, progression, surgical feasibility, comorbidities, treatment toxicity, or postoperative recovery. These observations support a longitudinal and adaptive multidisciplinary approach and may help identify clinical and organizational areas where perioperative care can be further improved. The study describes treatment feasibility without comparing the efficacy of different therapeutic strategies.

## 1. Introduction

The management of resectable NSCLC has evolved towards biologically informed multimodal strategies. Platinum-based chemotherapy remains an important component of perioperative chemo-immunotherapy, while validated molecular biomarkers identify patients eligible for targeted treatment. The CheckMate 816 study demonstrated that adding nivolumab to neoadjuvant chemotherapy improves pathological response and event-free survival without compromising the feasibility of surgical resection [1]. Subsequently, the KEYNOTE-671, AEGEAN, and CheckMate 77T studies expanded this paradigm through perioperative chemo-immunotherapy strategies, combining neoadjuvant treatment with continued immunotherapy after surgery [2,3,4]. Longer follow-up has strengthened the evidence for perioperative pembrolizumab: in KEYNOTE-671, five-year EFS was 49.9% versus 26.5%, and five-year OS was 64.6% versus 53.6%, with pembrolizumab versus placebo. Updated AEGEAN findings support sustained EFS benefit, whereas the interim OS estimate remains inconclusive (HR 0.89; 95% CI 0.70–1.14). At the same time, adjuvant targeted therapies have further expanded treatment options and made molecular characterization an integral part of the initial evaluation. Osimertinib and alectinib have demonstrated benefit in resected NSCLC harbouring *EGFR* mutations and *ALK* rearrangements, respectively [5,6]. More recently, LIBRETTO-432 demonstrated improved EFS with adjuvant selpercatinib after definitive treatment in *RET* fusion-positive NSCLC. In the primary stage II–IIIA population, two-year EFS was 92% versus 61% with placebo (HR 0.17; 95% CI 0.06–0.51) [7]. Defining the treatment strategy therefore requires an integrated assessment of molecular characteristics and PD-L1 expression, considered in parallel with clinical and radiological staging from the time of diagnosis. Early availability of this information can influence not only the choice of postoperative treatment but also the overall perioperative strategy. These advances have substantially changed the treatment paradigm for resectable NSCLC and have been incorporated into the most recent ESMO guidelines [8].

**Figure 1:**
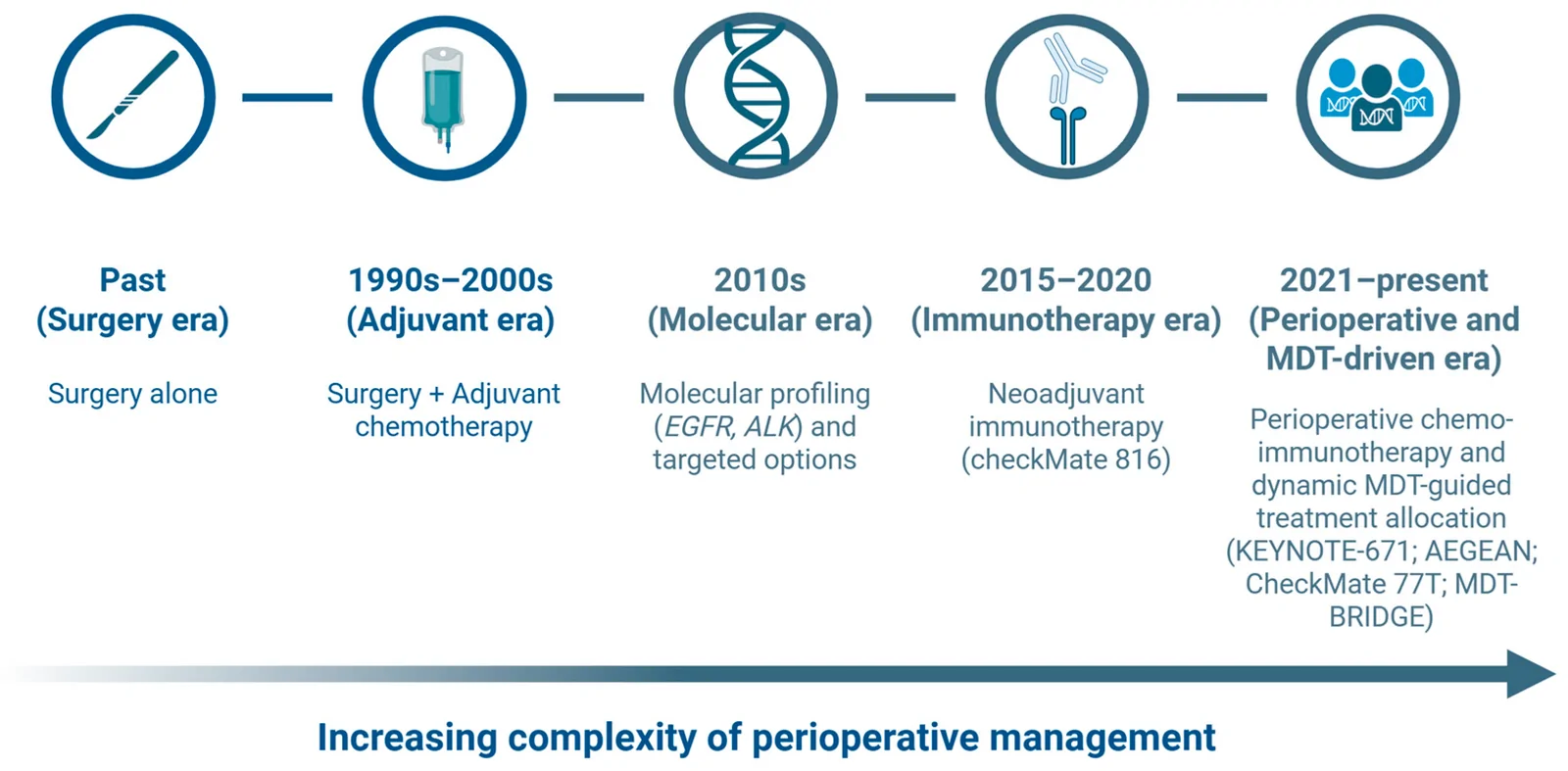
Evolution of the treatment paradigm in resectable NSCLC. Schematic representation of the transition from a surgery-centered approach to an increasingly personalized perioperative strategy integrating adjuvant chemotherapy, molecular profiling and targeted therapies, immunotherapy, and multidisciplinary decision-making. Contemporary management is characterized by increasing treatment complexity and by dynamic reassessment of resectability and therapeutic strategy throughout the perioperative pathway. The MDT-BRIDGE study [9] exemplifies this emerging MDT-driven approach, in which multidisciplinary reassessment after neoadjuvant therapy guides subsequent treatment allocation.

This evolution has made the decision-making process increasingly complex. Although the TNM stage remains fundamental, treatment selection today requires consideration of biological and molecular characteristics, mediastinal lymph node involvement, technical resectability, clinical and functional status, comorbidities, and the ability to complete postoperative treatment [8]. Stage II–III NSCLC, in fact, represents a heterogeneous population, in which patients with the same anatomical stage may present with different tumour burdens, lymph node patterns, and biological and clinical characteristics. Treatment decisions may therefore require reassessment throughout the therapeutic pathway according to treatment response, nodal status, surgical and pathological findings, and molecular information. Moreover, the expanding choice between neoadjuvant-only and perioperative immunotherapy strategies further increases the complexity of treatment sequencing and surgical integration [9,10]. Figure 1 summarizes this evolution, from the central role of surgery to a personalized, multidisciplinary perioperative model, in which the treatment strategy is redefined throughout the course of care based on the characteristics of the disease and the response to treatment.

This study aims to examine the major decision-making challenges in stage II–III NSCLC through a retrospective real-world analysis of consecutive patients discussed within our thoracic MDT, interpreted in the context of the available evidence. Specifically, we sought to identify the main decision points at which the initially considered therapeutic strategy was confirmed, modified, or could not be completed, and to explore the clinical factors underlying these changes.

## 2. Materials and Methods

### 2.1. Study Design, Population, and Data Sources

We conducted a single-centre, retrospective, descriptive observational study of 101 consecutive patients with NSCLC discussed within the thoracic multidisciplinary team (MDT) at Fondazione IRCCS San Gerardo dei Tintori, Monza, Italy, between 2023 and 2025. Patients were identified through MDT meeting records. The cohort included patients with clinical stage II–III disease at their initial MDT discussion and patients with pathological stage II–III disease discussed after surgical resection for consideration of adjuvant treatment and subsequent oncological management, including patients initially classified as clinical stage I. All consecutive patients meeting these inclusion criteria were included. No additional exclusion criteria were applied, and patients were not excluded because of missing molecular or clinical information.

MDT meeting records were the primary data source. Medical records were additionally reviewed when information was missing or incomplete in the MDT documentation. Follow-up information was collected up to December 2025.

The cohort was assembled to describe dynamic longitudinal decision-making trajectories within a single MDT rather than variability between different MDTs or treatment efficacy. The findings were interpreted in the context of the available literature.

### 2.2. MDT Organisation and Decision-Making

The thoracic MDT meets weekly and includes medical oncologists, pulmonologists, thoracic surgeons, radiologists, radiation oncologists, and pathologists. The minimum attendance requirement is at least one representative from each of these six specialties, although meetings usually involve at least two representatives per specialty. Treatment recommendations and any additional investigations requested are documented in the MDT meeting records. When disagreement or uncertainty prevents a shared treatment recommendation, the case is generally deferred for further diagnostic or clinical assessment and subsequently rediscussed within the MDT.

The analysis was organised around three main decision points: initial treatment selection, reassessment of resectability and operability after preoperative treatment, and postoperative assessment of recurrence risk and treatment feasibility.

### 2.3. Assessment of Neoadjuvant Candidacy

Potential candidacy for neoadjuvant treatment was assessed during contemporaneous MDT discussions using the information available at the time of clinical decision-making. For the present analysis, candidacy and the documented reasons for selecting an alternative initial treatment pathway were extracted from MDT meeting records rather than reconstructed using subsequent pathological findings or clinical outcomes. Patients initially classified as clinical stage I and included following pathological upstaging were not considered potential neoadjuvant candidates solely on the basis of their postoperative stage.

### 2.4. Molecular and Pathological Assessment

Molecular testing was performed on diagnostic biopsy material in patients considered for neoadjuvant treatment and on surgical specimens after pathological staging in patients undergoing upfront surgery. Available specimens included biopsies, cytological cell blocks and surgical resections.

DNA- and RNA-based targeted next-generation sequencing was performed on the Genexus Integrated Sequencer platform (Thermo Fisher Scientific, Carlsbad, CA, USA), using Oncomine Precision Assay panels (Thermo Fisher Scientific) with different configurations. DNA and RNA were extracted from formalin-fixed, paraffin-embedded material using the Maxwell CSC 16 platform and the corresponding CSC DNA FFPE and CSC RNA FFPE kits (Promega Corporation, Madison, WI, USA), and quantified using a Qubit 4 Fluorometer (Thermo Fisher Scientific). Tumour areas were selected by a pathologist and macrodissected as required. The assay configurations covered hotspot sequence variants in 45 or 48 genes, copy-number alterations in 14 genes, gene fusions involving 18 genes, and splice variants in three genes. The customised 48-gene configuration additionally included *KEAP1* and *STK11*. Sequencing data were analysed using Ion Torrent Genexus Software 6.8 (Thermo Fisher Scientific). Variant reporting thresholds differed between configurations, with reported allele-frequency cut-offs of >2% or >5%; a 5% limit of detection was specified for the customised 48-gene configuration. PD-L1 expression was assessed separately by immunohistochemistry using the 22C3 antibody clone (Dako/Agilent Technologies, Glostrup, Denmark) or the SP263 antibody clone (Ventana Medical Systems, Tucson, AZ, USA) on Dako Omnis or Ventana platforms, and reported as tumour proportion score (TPS).

Clinical staging was based on computed tomography (CT), supplemented by FDG-PET when clinically indicated, and reviewed within the MDT. Changes in clinical stage and treatment response were extracted from radiological reports and contemporaneous MDT assessments. No systematic retrospective RECIST reassessment was performed for this analysis. Disease stage was extracted as documented in the clinical and pathological records, using the TNM edition applicable at the time of assessment. Invasive mediastinal staging, including endobronchial ultrasound-guided transbronchial needle aspiration (EBUS-TBNA), was performed when clinically indicated according to the radiological and metabolic pattern of mediastinal lymph-node involvement. Pathological staging distinguished pTNM after upfront surgery from ypTNM after neoadjuvant treatment. pCR was recorded when explicitly documented in the surgical pathology report. Major pathological response (MPR) was not reported in this analysis. Details of invasive mediastinal staging were not uniformly available in the retrospective records.

### 2.5. Descriptive Analysis and Follow-Up

Categorical variables were summarised as counts and percentages, with denominators specified for each analysis. Missing information was reported where applicable. The analysis was descriptive, and no formal hypothesis testing was planned because of the exploratory nature of the study, the small sample size, and the heterogeneity of treatment pathways.

Oncological outcomes were described as documented progression or recurrence during the available follow-up up to December 2025. Events were identified from radiological reports and clinical assessments documented in MDT meeting records or medical records. Imaging findings were reviewed during routine MDT discussions with radiologist participation. No additional systematic retrospective review of imaging was performed specifically for this analysis. Crude event proportions were not interpreted as estimates of disease-free or progression-free survival.

## 3. Grey Areas and Unresolved Decision-Making in Perioperative NSCLC

### 3.1. Stage II NSCLC: Beyond Anatomical Staging

Stage II represents one of the contexts in which the application of current perioperative strategies remains less consistent. The TNM classification, indeed, encompasses different combinations of tumor extent and lymph node involvement, each associated with heterogeneous prognoses and risks of recurrence [11,12]. Consequently, being classified within the same anatomical stage does not necessarily identify patients with the same risk nor does it automatically imply the need for the same intensity of treatment. This heterogeneity is particularly relevant when deciding between upfront surgery and a preoperative systemic strategy, since the potential benefit of treatment intensification may differ substantially across stage II presentations.

Historically, in patients who underwent complete resection stage II NSCLC, platinum-based adjuvant chemotherapy has been the primary systemic treatment, with a relatively modest absolute benefit and a proportion of patients who, in clinical practice, are unable to complete the planned treatment [13,14]. The introduction of adjuvant immunotherapy has further complicated this scenario, but the available results are not entirely comparable. IMpower010 demonstrated a benefit in disease-free survival (DFS) with atezolizumab following adjuvant chemotherapy [15], while PEARLS/KEYNOTE-091 showed a benefit with pembrolizumab in the overall population, without a linear relationship with PD-L1 expression [16]. More recently, however, the randomized Phase III CCTG BR.31 trial failed to demonstrate an improvement in DFS with durvalumab compared to placebo in patients with completely resected *EGFR/ALK* wild-type NSCLC, neither in the primary population with PD-L1 ≥25% nor in subgroups defined by different PD-L1 expression thresholds [17]. These findings are particularly relevant in stage II disease, where anatomical staging encompasses patients with substantially different risks of recurrence and the indication for treatment intensification may therefore be less straightforward. These data indicate that neither anatomical stage nor a single biomarker is sufficient to define the optimal perioperative strategy in stage II disease. Patient selection and the optimal timing of systemic treatment therefore remain open questions.

Certain histopathological features may contribute to this stratification. In resected lung tumors, particularly adenocarcinomas, solid and micropapillary patterns have been associated with greater biological aggressiveness and a higher risk of recurrence [18,19,20]. Similarly, the discrepancy between clinical and pathological staging remains clinically relevant as unexpected lymph node upstaging at the time of surgery may reveal a disease that is biologically and prognostically different from what was initially diagnosed [21,22]. These factors may be particularly informative in stage II disease, where the indication for preoperative treatment may not be evident from clinical staging alone and additional risk features could help refine recurrence-risk assessment beyond anatomical staging.

In stage II disease, molecular characterization introduces an additional level of decision-making. In oncogene-addicted tumors, the availability of effective targeted strategies alters the relative value of the various perioperative options. In addition to the well-established data on *EGFR*, *ALK*, and *RET* in the adjuvant setting [5,6,7], NAUTIKA1 has explored the use of neoadjuvant targeted therapy in patients with resectable oncogene-driven NSCLC, including stage II disease [23]. The clinical challenge in stage II disease therefore extends beyond determining whether systemic treatment is indicated and includes defining its optimal timing and sequence in relation to surgery.

For tumors without actionable alterations, the ability of currently available biomarkers to individually guide treatment decisions remains more limited. PD-L1 is an established parameter, but the inconsistent results from adjuvant and perioperative studies highlight its limitations as a standalone decision-making biomarker [15,16,17,24]. Co-mutations in *STK11* and *KEAP1*, particularly in *KRAS*-mutated tumors, represent a possible additional level of biological stratification: they have been associated with reduced sensitivity to PD-(L)1 inhibition and, in the resectable setting, with different outcomes following neoadjuvant immunotherapy [25,26]. However, their value remains primarily prognostic and does not currently allow for the definition of validated alternative perioperative strategies. Accordingly, in stage II disease, these biomarkers cannot currently be used in isolation to determine whether a patient should undergo upfront surgery or receive preoperative systemic treatment.

Precision oncology extends beyond PD-L1 expression and individual actionable drivers. Comprehensive genomic profiling can identify co-occurring mutations, copy-number alterations and gene fusions, although a single sample may incompletely capture intratumoural heterogeneity and clonal evolution. TRACERx has demonstrated the relevance of subclonal architecture and genomic instability to relapse in resected NSCLC [27]. Transcriptomics, epigenomics, proteomics, metabolomics and characterisation of the tumour immune microenvironment offer complementary perspectives on tumour biology [28]. However, integrated multiomic profiling does not currently have an established role in routine perioperative treatment selection and requires prospective validation of clinical utility. Pharmacogenomic approaches may further contribute to understanding interindividual variability in treatment efficacy and toxicity, while functional drug-sensitivity platforms, including patient-derived organoids, may provide complementary information on tumour-specific therapeutic vulnerabilities. However, these approaches remain investigational in perioperative NSCLC and currently have no established role in routine treatment selection.

A potentially more dynamic approach involves the assessment of minimal residual disease (MRD) using circulating tumor DNA (ctDNA). Postoperative ctDNA detection has emerged as a strong prognostic marker, with persistence or reappearance after resection identifying patients at substantially higher risk of recurrence [29,30]. Beyond postoperative MRD assessment, longitudinal ctDNA monitoring throughout the perioperative pathway may provide complementary information on treatment response and residual disease, potentially enabling a more individualized approach to treatment escalation or de-escalation [29]. This strategy may be particularly relevant in stage II disease, where anatomical staging alone encompasses markedly heterogeneous recurrence risks and where a dynamic biomarker could potentially refine the selection of patients requiring treatment intensification. However, despite its biological and prognostic potential, the use of ctDNA to directly guide perioperative treatment decisions remains investigational and requires prospective validation.

Consequently, stage II NSCLC should not be interpreted as a single decision-making category. The clinical challenge is to recognize, within the same anatomical stage, different risk profiles and therapeutic needs by integrating anatomical staging, pathological and molecular characteristics, patient fitness, and, potentially, dynamic biomarkers such as ctDNA. In this context, the central multidisciplinary question is not simply whether systemic treatment is indicated, but also whether an individual patient is better served by upfront surgery followed by risk-adapted postoperative treatment or by an intensified preoperative/perioperative strategy.

### 3.2. Stage III NSCLC: Defining and Reassessing Resectability

In stage III, one of the main areas of uncertainty concerns the very definition of resectability. Operability and resectability must be considered separately: the former primarily concerns the patient’s ability to tolerate a surgical procedure, while the latter depends on the technical and oncological feasibility of achieving a complete resection. This distinction is particularly important in locally advanced tumors, where the T-stage, location, and extent of mediastinal involvement, as well as the need for complex resections, can lead to differing assessments even among expert centers.

The recent multisociety consensus coordinated by the EORTC Lung Cancer Group proposed a standardization of the concept of technical resectability, identifying the characteristics of N2 disease as one of the main determinants of the surgical decision [31]. Non-bulky single-station N2 involvement may be compatible with a surgical strategy, whereas bulky or infiltrative N2 involvement, extensive multistation involvement, and N3 generally indicate non-resectability [31]. The document also explicitly emphasizes that the definition of resectability must be derived from the assessment of experienced thoracic surgeons within the context of the MDT.

The need and difficulty of standardization are confirmed by studies that have directly assessed inter-specialist agreement. In the international survey by Houda et al., agreement was high in cases that were clearly resectable or non-resectable but decreased in intermediate anatomical configurations [32]. Similarly, the evaluation of clinical cases by a European multidisciplinary panel showed a greater propensity for surgery in cases of non-bulky, single-station N2 disease and a progressive shift toward non-surgical strategies in the presence of bulky N2, multistation N2, and N3 disease [33]. The concept of borderline resectability therefore identifies not an independent TNM category, but a decision-making zone in which anatomy, the likelihood of complete resection (R0), surgical risk, and the center’s experience must be considered collectively.

The availability of neoadjuvant chemoimmunotherapy has made this distinction even more relevant. Meta-analyses and real-world data show that a proportion of patients with locally advanced disease can undergo resection after induction therapy [34,35]. However, post-induction evaluation introduces a new source of uncertainty: radiological response and pathological response do not necessarily coincide. Fibrosis, necrosis, and immune infiltration can result in modest dimensional reduction despite marked regression of the viable tumor, limiting the value of radiological criteria alone in surgical decision-making.

Re-evaluation should therefore focus not only on the extent of the response but, above all, on the persistence of a realistic possibility of R0 resection and on the feasibility of the planned procedure. This aspect takes on particular importance when highly complex procedures are required. Pneumonectomy following neoadjuvant therapy may be feasible in carefully selected patients, but it carries a different risk profile compared to parenchyma-sparing resections [36]. The role of salvage surgery following definitive chemoradiotherapy is even more selective, with the available evidence deriving primarily from retrospective series at high-volume centers [37,38,39].

Resectability in stage III should therefore be considered dynamic and reassessed throughout treatment according to the likelihood of achieving an R0 resection and the expected surgical risk.

### 3.3. MDT and Variability in Complex Clinical Decisions

The benefits of multidisciplinary management in lung cancer are supported by observational studies, systematic reviews, and meta-analyses that have demonstrated associations with more comprehensive staging, better adherence to treatment pathways, and, in some case series, better outcomes [40,41,42,43,44]. However, in patients with locally advanced NSCLC, the most important issue is not simply the presence of an MDT, but the team’s ability to formulate a strategy when the available evidence allows for more than one reasonable option.

This limitation emerges from studies that have directly assessed the reproducibility of multidisciplinary decisions. Hoeijmakers et al. documented significant variability among different tumor boards in staging and treatment recommendations formulated for the same cases of locally advanced NSCLC [45]. Mainguene et al. similarly reported only moderate agreement in the choice between surgical and nonsurgical strategies in patients with stage IIIA/B-N2 disease [46]. Discrepancies are greatest precisely in intermediate cases, where mediastinal extension, lymph node burden, and the likelihood of complete resection do not allow for an unambiguous classification.

Even adherence to guidelines does not necessarily equate to the appropriateness of an individual decision. Vinod et al. have shown that recommendations formulated in a multidisciplinary setting may deviate from guideline-based recommendations, especially in elderly patients, those with impaired performance status, or those with locally advanced disease [47]. In standardized cases, the MDT therefore facilitates the application of evidence, while in gray areas its primary function is to interpret evidence considering clinical characteristics that are underrepresented in clinical trials.

This function has become even more relevant with contemporary perioperative strategies. Recent expert consensus statements emphasize that the choice between upfront surgery, neoadjuvant therapy, perioperative strategies, and non-surgical approaches requires a joint assessment of resectability, mediastinal staging, molecular profile, and patient characteristics [48]. The composition and experience of the team can also influence the decision, especially when highly specialized surgical skills are required or when multiple potentially appropriate treatment options coexist [49].

**Figure 2:**
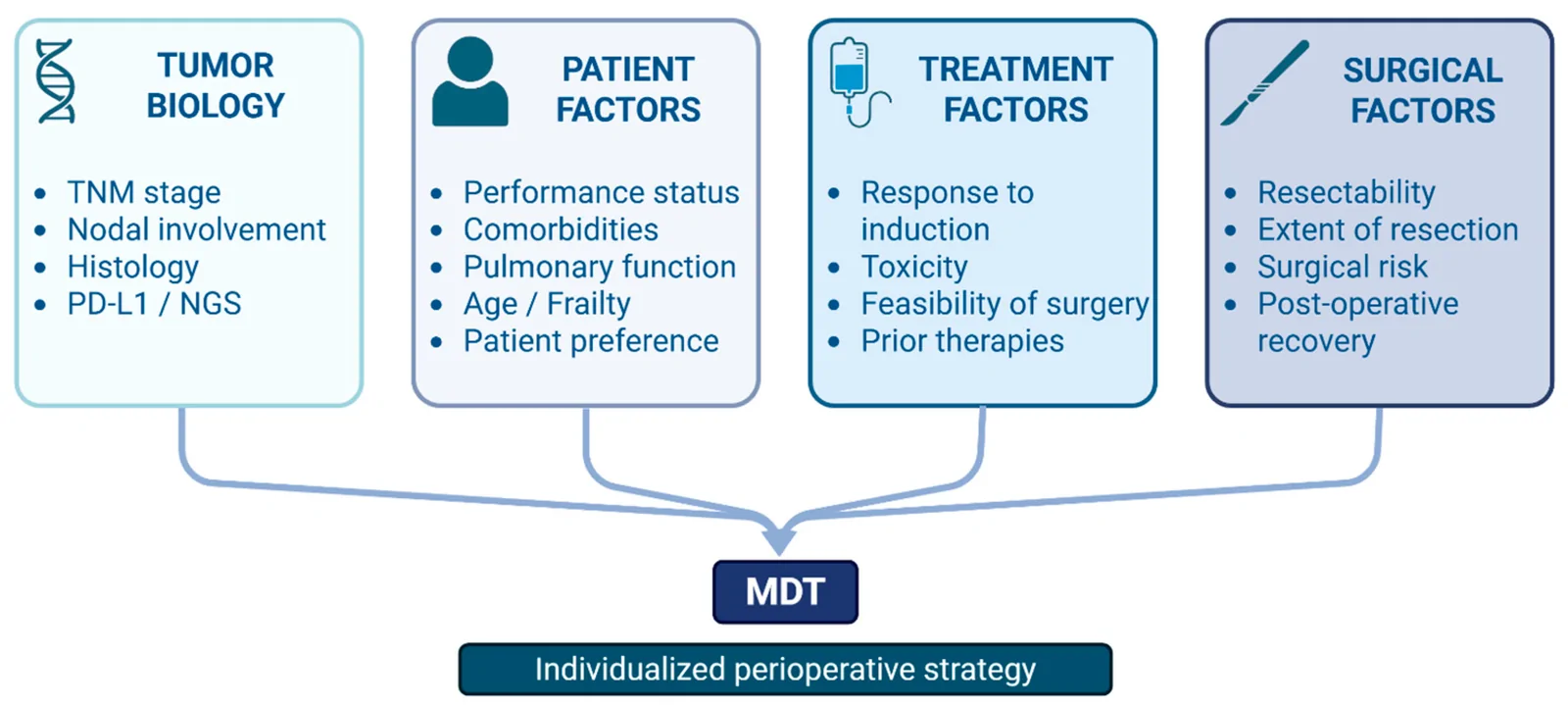
Multidimensional determinants of MDT decision-making in perioperative NSCLC. Perioperative treatment allocation results from the integration of tumour-related characteristics, patient fitness and preferences, response and tolerability to systemic treatment, and technical surgical considerations. Rather than being determined by anatomical stage alone, these factors are dynamically reassessed within the MDT to define an individualized perioperative strategy.

Variability among MDTs should therefore not be interpreted solely as a lack of standardization, but also as a possible indicator of areas in which the available evidence lacks sufficient granularity to guide individual cases. In these situations, the treatment strategy is derived from the dynamic integration of the biology and extent of the disease, patient characteristics, response to treatment, and surgical factors (Figure 2). The MDT thus provides the framework within which these dimensions are synthesized into an individualized perioperative strategy.

## 4. MDT Real-World Experience

### 4.1. Patient Characteristics and Clinical-Biological Heterogeneity

The study population comprised 101 patients, of whom 61 were men (60.4%) and 40 were women (39.6%). Adenocarcinoma was the most common histological type, accounting for 76 cases (75.2%), followed by squamous cell carcinoma in 20 cases (19.8%); the remaining five cases comprised less common histologies.

The performance status was ECOG 0 in 30 patients (29.7%), ECOG 1 in 39 (38.6%), ECOG 2 in 12 (11.9%) and ECOG 3 in 3 (3.0%); this data was not available for 17 patients (16.8%). Although the majority of the cohort had a PS ECOG status of 0–1, the cohort included a clinically significant proportion of patients with reduced fitness, a factor that substantially influenced both access to multimodal treatments and eligibility for surgery.

Following reconciliation of clinical and pathological data, 41 patients were classified as stage II (40.6%) and 60 as stage III (59.4%). The discrepancy between clinical and pathological staging was clinically significant: four patients initially classified as stage I were found to be stage II after resection, whilst a further case with pT4N0 was reclassified as stage IIIA. These cases demonstrate how pathological staging can provide information not available at the time of the initial decision, thereby altering subsequent prognostic stratification and, potentially, the postoperative strategy [21,22].

The availability of biomarkers was not uniform. PD-L1 was available in 76 out of 101 patients (75.2%), whilst the molecular profile obtained via NGS was available in 57 out of 101 (56.4%). Alterations in *EGFR*, *ALK*, *KRAS* and other genes potentially relevant from a prognostic or therapeutic perspective were documented. In the current perioperative paradigm, in which molecular characterisation can influence both the postoperative strategy and the overall treatment sequence [5,6,7,8,50], the timely availability of such information is significant not only from a biological perspective but also from an organisational one. The main clinical, pathological and molecular characteristics of the population, together with the distribution of the main treatment pathways, are summarised in Table 1.

### 4.2. First Decision Point: Selection of Preoperative Treatment and Reassessment After Induction

The first clinically significant decision concerned the choice between upfront surgery and pre-operative treatment. Thirty patients were considered potential candidates for non-targeted neoadjuvant treatment during contemporaneous MDT discussions. Of these, 20 received treatment and ten followed an alternative initial pathway. Three additional patients received neoadjuvant alectinib within the ALNEO trial.

Of these, 20/30 (66.7%) received non-targeted neoadjuvant treatment, whilst 10/30 (33.3%) were placed on a different initial treatment pathway. Among the ten patients who did not receive neoadjuvant therapy, in 5 out of 10 cases (50.0%), the MDT had deliberately opted for upfront surgery due to favourable resectability and a limited disease burden. In 3 out of 10 patients (30.0%), preoperative treatment was not administered because of inadequate diagnostic material or an incomplete preoperative assessment; two of these patients subsequently received definitive chemoradiotherapy, while one underwent upfront surgery. In the remaining 2 out of 10 patients (20.0%), comorbidities or contraindications precluded neoadjuvant treatment; one patient subsequently underwent upfront surgery, while the other received definitive chemoradiotherapy.

The non-administration of neoadjuvant therapy did not, therefore, constitute a uniform category. It encompassed an intentional clinical decision by the MDT, a limitation in the diagnostic pathway, and a limitation related to patient characteristics. This distinction is relevant when interpreting real-world practice, as a deviation from the theoretically applicable pathway does not necessarily equate to a lack of appropriateness.

Taking the entire population into account, 23 out of 101 patients (22.8%) received preoperative systemic treatment. Of these, 20 received non-targeted neoadjuvant treatment, whereas 3 *ALK*-positive patients received neoadjuvant alectinib within the ALNEO trial [50]. Among the 20 patients receiving non-targeted treatment, 14 received chemo-immunotherapy or investigational immunotherapy-based regimens and 6 received chemotherapy without immunotherapy. The distribution of preoperative strategies and subsequent postoperative treatments in resected patients is shown in Table 2.

Following pre-operative treatment, 17 out of 23 patients (73.9%) went on to undergo surgery, whilst 6 out of 23 (26.1%) did not undergo resection. The reasons were a shift from initially considered resectable or borderline-resectable disease to disease deemed unresectable at post-treatment reassessment in 3 patients (13.0%), disease progression during treatment in 2 (8.7%), and toxicity in 1 (4.3%). These scenarios represent clinically distinct conditions that include patients with disease progression for whom pre-operative treatment enabled the identification of a biologically aggressive disease prior to major surgery; patients in whom post-treatment reassessment indicated that an R0 resection was no longer considered realistically achievable despite preoperative therapy; the patient with treatment-related toxicity for whom the surgical strategy was no longer considered clinically feasible.

At the same time, profound responses capable of favourably altering the course of treatment have been observed. A patient with cT4N2M0, stage IIIB adenocarcinoma, treated as part of the NEOCOAST-2 trial, achieved downstaging to ypT2bN0 and subsequently underwent R0 resection. A patient with cT2bN1 disease, treated as part of the AEGEAN Early Access Programme, achieved a complete pathological response (ypT0N0) and subsequently completed postoperative durvalumab.

The three *ALK*-positive patients treated with alectinib as part of the ALNEO trial [50] were also particularly informative. Despite sharing the same oncogenic driver and therapeutic strategy, three distinct trajectories were observed: pCR, lymph node downstaging from N2 to N1, and persistence of N2 disease with subsequent suspected bone progression. These cases demonstrate how molecular characterisation can guide treatment selection without completely predetermining the individual response.

Among the 17 patients who underwent surgery following pre-operative treatment, the treatment pathway did not necessarily conclude with resection. Ten patients (58.8%) continued with post-operative systemic treatment: three with alectinib, four with durvalumab, one with pemetrexed and pembrolizumab, one with pembrolizumab and sacituzumab tirumotecan, and one with rilvegostomig. Furthermore, 8 out of 17 patients (47.1%) received consolidation radiotherapy, four of whom also received this in combination with postoperative systemic treatment. These data highlight the heterogeneity of the perioperative regimens actually implemented in clinical practice.

### 4.3. Second Decision Point: Resectability, Operability, and Alternative Locoregional Strategies

Overall, 66 of 101 patients (65.3%) underwent surgical resection, of whom 49 underwent upfront surgery and 17 underwent surgery following preoperative treatment. The remaining 35 of 101 patients (34.7%) did not undergo surgery.

Notably, the absence of surgery did not necessarily correspond to oncological unresectability. In the six patients described above, resection was not performed after preoperative treatment because disease was deemed unresectable at post-treatment reassessment, because of disease progression, or because of treatment-related toxicity; in the remaining patients who did not undergo surgery, the decision was primarily influenced by clinically significant comorbidities and/or impaired respiratory function. The population that did not undergo surgery therefore encompassed different conditions, in which oncological unresectability and medical inoperability were not necessarily synonymous.

This distinction is particularly relevant in stage III, where the technical feasibility of achieving an R0 resection must be considered separately from the patient’s ability to tolerate the procedure. Analyses of the multidisciplinary definition of resectability confirm that intermediate cases, especially those with N2 involvement or T3–T4 disease, are characterized by greater variability in decision-making [31,32,33].

The role of alternative locoregional strategies is clearly evident in our case series. Indeed, 25 of 35 patients who did not undergo surgery (71.4%) received radiation therapy. In most cases, therefore, the decision not to perform resection did not mean forgoing locoregional control, but rather selecting an alternative strategy deemed more appropriate given the patient’s oncological, functional, and clinical characteristics. This finding also highlights the role of the radiation oncologist within the multidisciplinary decision-making process.

Even in technically resectable patients, the extent of the necessary surgery could substantially alter the risk–benefit ratio. A representative case involved a patient with stage IIIA cT4N1M0 squamous cell carcinoma who underwent left pneumonectomy after cisplatin-gemcitabine and subsequently developed complications including pancytopenia, deep vein thrombosis, and acute renal failure. This case highlights that technical resectability, operability, and overall clinical appropriateness are not interchangeable concepts. The literature confirms that extensive resections following induction therapy can be performed in carefully selected patients but require a thorough assessment of functional and perioperative risks [36].

Therefore, the decision to proceed with surgery following neoadjuvant treatment represented a second true decision point. It is not merely an assessment of the technical feasibility of resecting the tumor, but it also requires a comprehensive reevaluation in which treatment response, the likelihood of an R0 resection, the anticipated surgical extent, comorbidities, and functional reserve all factored into the choice between surgery and alternative locoregional strategies.

### 4.4. Third Decision Point: Pathological Staging, Postoperative Treatment, and Oncological Outcomes

Surgical resection introduced an additional opportunity for reassessment. The definitive pathological findings could alter the risk assessment and treatment recommendations, while the postoperative course and recovery could tangibly affect the feasibility of treatment.

In our series, we observed several instances of unexpected pathological upstaging. One patient initially classified as cT2aN0 was found to be pT3N0 after resection and subsequently underwent adjuvant chemotherapy followed by atezolizumab. A second patient, initially classified as cT1bN0, was found to have pT1cN1 disease after resection. The patient was subsequently considered ineligible for adjuvant therapy and later developed cerebral recurrence. In another case, despite a negative systematic mediastinal staging result by endobronchial ultrasound (EBUS), the definitive pathological examination revealed occult pN2. These scenarios are consistent with the literature on mediastinal upstaging [21,22] and demonstrate how pathological staging can redefine not only the prognosis but also the subsequent treatment sequence.

Of the 66 patients who underwent surgery, 30 (45.5%) received postoperative systemic treatment, while 36/66 (54.5%) did not receive adjuvant therapy. Of the latter group, 18 out of 36 (50.0%) were not treated due to comorbidities or clinical frailty, 7 out of 36 (19.4%) due to postoperative complications or insufficient functional recovery, 6 out of 36 (16.7%) due to refusal of treatment, and 5 out of 36 (13.9%) because the risk of recurrence was considered low or the risk–benefit ratio of the treatment was unfavorable. Overall, therefore, in 25 out of 36 patients (69.4%), the failure to administer adjuvant therapy was primarily attributable to comorbidities, frailty, or postoperative complications, highlighting the potential gap between theoretical oncological indications and the actual feasibility of treatment.

At the opposite extreme, a patient with pT2bN0 adenocarcinoma, 1% PD-L1 expression, and no actionable drivers, managed through surveillance without adjuvant therapy, maintained prolonged disease control. Although this does not allow for causal inferences, the case underscores the mirror-image problem of overtreatment and the need to balance the risk of recurrence with the expected absolute benefit and potential toxicity.

Molecular characterization has also directly altered certain postoperative management pathways. Three patients with stage II *EGFR*-mutated adenocarcinoma received adjuvant osimertinib, confirming that molecular profiling is now an integral part of defining the perioperative strategy [5]. Looking ahead, ctDNA could contribute to further risk stratification as the persistence or reappearance of ctDNA after resection is associated with a higher risk of recurrence [30], while longitudinal monitoring could provide complementary information on response and residual disease [29]. However, its use to directly guide escalation or de-escalation strategies remains experimental [29].

During follow-up, 34 of 101 patients (33.7%) developed documented progression or recurrence, while in the remaining 67 of 101 (66.3%), no event was documented in the available records up to December 2025. These findings describe recorded events within a heterogeneous cohort and should not be interpreted as formal estimates of DFS or PFS, given the variable duration and completeness of follow-up.

The persistence of a significant cancer risk is supported by real-world evidence. In the RESECT study, post-resection outcomes progressively worsened as the stage increased [51]; in the post-neoadjuvant setting, Li et al. identified the percentage of viable residual tumor and the pathological lymph node burden as determinants of risk and recurrence patterns [52]. Even a complete pathological response does not equate to the absolute elimination of risk, as suggested by the recurrences documented by Fang et al. in patients with pCR following neoadjuvant immunochemotherapy [53].

Taken together, the results allow for the identification of three main points at which the treatment pathway could diverge from the initially hypothesized strategy: selection of preoperative treatment, access to surgery, and feasibility of postoperative treatment. The size of each population and the main reasons underlying the changes in the treatment pathway are summarized in Table 3.

Beyond the aggregate data, certain patients particularly highlight the complexity of the decision-making process. Downstaging and pCR could support the surgical pathway, while disease progression during induction therapy, unresectability at post-treatment reassessment, pathological upstaging, surgical morbidity, and reduced postoperative fitness could lead to a redefinition of the initial strategy. The main challenging scenarios observed in our cohort and their implications for multidisciplinary decision-making are summarized in Table 4.

Overall, our experience shows that the treatment pathway for stage II–III NSCLC is rarely linear. The three decision points identified, selection of preoperative treatment, eligibility for surgery, and determination of the postoperative strategy, are not independent phases, but rather successive stages of the same adaptive process. The ability to reassess the strategy at each of these junctures based on tumor biology, response, resectability, operability, and patient fitness is therefore one of the central elements of real-world multidisciplinary management.

## 5. Discussion

This real-world analysis of 101 patients with stage II–III NSCLC demonstrates that the contemporary management of resectable or potentially resectable disease is difficult to fit into a strict treatment sequence. The most significant finding from our experience is the gap that can arise among theoretical eligibility, the strategy defined by the MDT, the treatment actually administered, and the possibility of completing the entire course of treatment.

This becomes apparent as early as the selection of neoadjuvant therapy. Of the 30 patients considered potential candidates for non-targeted neoadjuvant treatment, ten followed an alternative initial pathway. In half of these cases, upfront surgery was a deliberate choice by the MDT due to favourable resectability and a limited disease burden; in the other patients, the reasons included inadequate diagnostic material, incomplete pre-operative assessment or clinical contraindications. These scenarios distinguish between appropriate clinical variability and diagnostic, organisational or patient-related limitations. In particular, within the current biomarker-driven paradigm, the adequacy of the sample and the timely availability of molecular profiling become an integral part of the quality of the treatment pathway.

Of the 23 patients treated, 17 (73.9%) underwent resection, while 6 did not proceed to surgery because disease was deemed unresectable at post-treatment reassessment, disease progression, or treatment-related toxicity. The reasons for not proceeding with surgery represent clinically distinct scenarios. In fact, progression may indicate aggressive tumour biology at an early stage; disease deemed unresectable at reassessment reflects the inability to identify a realistic R0 surgical option despite preoperative treatment, while toxicity may alter operability regardless of tumour response. The pre-operative phase therefore also serves a biological and clinical selection function.

At the same time, cases with pCR and significant downstaging demonstrate the potential of induction therapy to favourably alter resectability. The heterogeneity observed in *ALK*-positive patients treated with alectinib, however, highlights that different pathological outcomes are possible even in the presence of the same driver and the same treatment strategy. These differences cannot be attributed to specific biological mechanisms, as co-occurring alterations, intratumoural heterogeneity and the immune microenvironment were not systematically investigated in this cohort. They nevertheless support individual reassessment rather than an assumption of uniform response based on driver status alone. This supports a dynamic conception of resectability, which must always be distinguished from the patient’s operability. Germline pharmacogenomics may help investigate interindividual differences in drug efficacy and toxicity, whereas functional models, including patient-derived organoids, may explore tumour-specific drug sensitivity and resistance. Their clinical translation is limited by the need for validation, assay standardisation and adequate representation of tumour-microenvironment interactions. Neither approach is an established requirement for perioperative treatment selection in NSCLC or was used in the present analysis [54,55].

This distinction is clear in the 35 patients who did not undergo surgery, in whom comorbidities and reduced respiratory function frequently represented the main barrier to surgery. The fact that 25 out of 35 (71.4%) received radiotherapy further demonstrates that the decision not to perform resection did not equate to abandoning locoregional control, but rather to the choice of an alternative strategy deemed more appropriate. Similarly, in patients undergoing major resections, technical feasibility must be balanced against surgical risk and overall oncological benefit [31,32,33,36].

A further critical point arises following surgery. Cases of pathological upstaging confirm that an initial underestimation can alter the postoperative strategy [21,22], whilst the possibility of administering adjuvant therapy depends heavily on clinical conditions. Of the 36 patients who underwent surgery but did not receive postoperative therapy, 25 (69.4%) were not treated primarily due to comorbidities, frailty or postoperative complications. The absence of adjuvant therapy cannot therefore be automatically interpreted as undertreatment, but must be distinguished from clinical impossibility of treatment, patient refusal or an unfavourable risk–benefit assessment. The negative result of the BR.31 trial [17] further reinforces the need to avoid indiscriminate intensification of treatment.

Oncological outcomes confirm that the residual risk remains significant. Documented progression or recurrence was observed in 34 out of 101 patients (33.7%). Although this does not represent a formal estimate of DFS, this finding is consistent with real-world evidence. Indeed, in the RESECT study, outcomes worsened as the stage increased [51], whilst Li et al. demonstrated that residual viable tumour and pathological lymph node burden following neoadjuvant therapy are associated with the risk of recurrence [52]. Even pCR does not eliminate this risk, as demonstrated by the recurrences observed by Fang et al. [53].

Longitudinal ctDNA assessment may complement perioperative risk evaluation by providing information on molecular residual disease, treatment response and molecular relapse before radiological progression. Serial profiling may also help investigate tumour evolution. Interpretation depends on assay sensitivity, sampling time and tumour shedding; a negative result does not exclude residual disease. Prognostic associations should therefore be distinguished from evidence that ctDNA-guided treatment changes improve outcomes. Treatment escalation or de-escalation based on ctDNA remains investigational [29,30].

Overall, our data suggest that the therapeutic variability observed in clinical practice does not necessarily equate to inappropriate treatment. Some deviations from the theoretically expected course reflect a correct individualisation of the strategy, whilst others identify areas with potential for improvement, in particular the adequacy of diagnostic material and the timeliness of molecular characterisation. The value of the MDT therefore lies in the ability to reassess the strategy over time based on response, progression, toxicity, resectability, pathological findings, and post-operative recovery [9,40,41,42,43,44,45,46,47,48,49]. Increasing molecular complexity may warrant additional input from molecular pathologists and genomic medicine specialists within multidisciplinary decision-making.

Our analysis has the limitations inherent in a single-centre retrospective series: a relatively small sample size, heterogeneity of treatments, rapid evolution of standards during the period under consideration, and incomplete availability of certain clinical, molecular and follow-up variables. The inclusion of patients identified at different points in the pathway, either through clinical stage II–III disease or postoperative pathological stage II–III disease, should also be considered when interpreting the cohort. Furthermore, the classification of outcomes as ‘documented progression/recurrence’ and ‘no documented events’ is purely descriptive. Although potential candidacy for neoadjuvant treatment was assessed during contemporaneous MDT discussions rather than reconstructed retrospectively, incomplete documentation of the decision-making rationale and retrospective interpretation of treatment trajectories remain possible sources of bias.

Despite these limitations, our real-world analysis complements evidence from registration trials by describing treatment feasibility across the entire perioperative pathway, including where planned strategies are modified and the clinical reasons underlying these changes. From this perspective, the main challenge in perioperative management is not merely choosing the treatment but correctly adapting the sequence of treatments to the progression of the disease and the patient’s condition.

## 6. Conclusions

The management of stage II–III NSCLC is now characterised by an increasing range of neoadjuvant, perioperative, adjuvant, surgical, radiotherapy and biomarker-driven strategies. However, our real-world experience shows that the strategy implemented may differ significantly from that theoretically envisaged on the basis of anatomical stage alone.

The main points of divergence observed in our cohort concerned selection for pre-operative therapy, eligibility for surgery and the actual possibility of completing post-operative treatment. At each of these stages, tumour-related and patient-related factors (resectability, operability, response, progression, toxicity, comorbidities, postoperative recovery, adequacy of diagnostic assessment and patient preferences) could alter the initially planned course of treatment. At the same time, the progression or recurrence following either upfront surgery or neoadjuvant therapy confirms the persistence of a clinically significant oncological risk despite strategies aimed at radical treatment.

These data support a longitudinal and adaptive decision-making model, in which the MDT does not represent a single point in the management process, but rather the context in which the strategy is periodically reassessed based on new information that becomes available.

Therapeutic variability should therefore not automatically be interpreted as inappropriate as some of it reflects a necessary individualised approach, whilst other deviations may highlight areas for improvement in the diagnostic and organisational pathway. Future research should determine whether integrating longitudinal molecular measurements with anatomical staging, pathological response and clinical fitness improves perioperative decision-making. Improved risk stratification should be distinguished from evidence that biomarker-directed treatment escalation or de-escalation improves patient outcomes.

The main objective of perioperative management is therefore not merely to identify the correct treatment, but to construct and adapt, over time, the most appropriate treatment sequence for the individual patient.

## Figures and Tables

**Table 1:** Baseline demographic, clinical, pathological, molecular, and treatment characteristics of the overall study population (*N* = 101). Data are presented as number and percentage unless otherwise specified. Abbreviations: ECOG PS, Eastern Cooperative Oncology Group Performance Status; NGS, next-generation sequencing; and PD-L1, programmed death-ligand 1. Treatment pathway rows are not mutually exclusive: patients undergoing surgery after preoperative treatment are also included in the preoperative systemic treatment category.

Characteristic	Patients, n (%)	Characteristic	Patients, n (%)
Sex:		Final disease stage:	
Male	61 (60.4)	Stage II	41 (40.6)
Female	40 (39.6)	Stage III	60 (59.4)
Histology:		Biomarker assessment:	
Adenocarcinoma	76 (75.2)	PD-L1 available	76 (75.2)
Squamous cell carcinoma	20 (19.8)	NGS available	57 (56.4)
Other histologies	5 (5.0)		
ECOG performance status:		Treatment pathway:	
ECOG PS 0	30 (29.7)	Preoperative systemic treatment	23 (22.8)
ECOG PS 1	39 (38.6)	Upfront surgery	49 (48.5)
ECOG PS 2	12 (11.9)	Surgery after preoperative treatment	17 (16.8)
ECOG PS 3	3 (3.0)	No surgery	35 (34.7)
Missing	17 (16.8)		

**Table 2:** Preoperative systemic strategies administered in the study cohort and subsequent postoperative treatment approaches among patients undergoing surgical resection. Among the 17 patients undergoing surgery after preoperative treatment, 10/17 (58.8%) received postoperative systemic therapy and 8/17 (47.1%) received consolidative radiotherapy; four patients received both modalities. Abbreviations: ALNEO, neoadjuvant alectinib trial.

Preoperative Treatment Strategy	Patients, n (%)	Postoperative Treatment Strategy Among Resected Patients
Chemo-immunotherapy or investigational immunotherapy-based strategies	14 (60.9)	Durvalumab-, pembrolizumab-, or rilvegostomig-based treatment according to the perioperative strategy
Platinum-based chemotherapy without immunotherapy	6 (26.1)	Postoperative treatment according to pathological findings and clinical indication
Alectinib within the ALNEO trial	3 (13.0)	Alectinib
Total	23 (100)	-

**Table 3:** Summary of the major multidisciplinary decision points identified along the real-world treatment pathway. The table illustrates the proportion of patients completing or deviating from the initially considered therapeutic strategy and the main clinical, disease-related, and organizational factors underlying treatment modification.

Decision Point	Population	Treatment Pathway	Main Reasons for Pathway Modification
Selection for neoadjuvant treatment	30 patients considered potential candidates for non-targeted neoadjuvant treatment	20 (66.7%) received non-targeted neoadjuvant treatment; 10 (33.3%) followed an alternative initial treatment pathway	Upfront surgery for favorable resectability/limited disease burden: 5/10 (50.0%); inadequate diagnostic tissue or incomplete preoperative assessment: 3/10 (30.0%); comorbidities/contraindications: 2/10 (20.0%)
Reassessment after preoperative treatment	23 treated patients	17 (73.9%) underwent surgery; 6 (26.1%) did not	Disease deemed unresectable at post-treatment reassessment: 3/23 (13.0%); disease progression: 2/23 (8.7%); treatment-related toxicity: 1/23 (4.3%)
Overall surgical decision	101 patients	66 (65.3%) underwent surgery; 35 (34.7%) did not	Outside the post-neoadjuvant setting, major limiting factors included comorbidities and/or impaired respiratory function
Locoregional management in non-operated patients	35 non-operated patients	25/35 (71.4%) received radiotherapy	Alternative locoregional strategy when surgery was not feasible or appropriate
Postoperative systemic treatment	66 operated patients	30 (45.5%) received postoperative systemic treatment; 36 (54.5%) did not	Comorbidities/frailty: 18/36 (50.0%); postoperative complications/inadequate recovery: 7/36 (19.4%); patient refusal: 6/36 (16.7%); low recurrence risk or unfavorable risk–benefit assessment: 5/36 (13.9%)

**Table 4:** Representative challenging scenarios encountered during multidisciplinary management of the study population. These cases illustrate how treatment response, tumor biology, pathological staging, surgical complexity, and patient fitness may modify the therapeutic pathway at different decision points.

Clinical Challenge	Representative Finding	MDT/Clinical Implication
Major downstaging after induction treatment	cT4N2M0 stage IIIB → ypT2bN0 after NEOCOAST-2 treatment	Treatment response converted a challenging presentation into an R0 surgical pathway
Pathological complete response	cT2bN1 → ypT0N0 after an AEGEAN-based strategy	Profound pathological response following preoperative chemo-immunotherapy
Heterogeneous response in oncogene-driven disease	Three *ALK*-positive patients treated with alectinib achieved pCR, N2 → N1 downstaging, and persistent N2 disease, respectively	The same molecular driver and treatment strategy did not result in a uniform biological response
Progression during neoadjuvant treatment	Disease progression prevented surgery in 2 patients	Preoperative treatment may identify biologically aggressive disease before major surgery
Unresectability at post-treatment reassessment after induction	Three patients were deemed unresectable at post-treatment reassessment	Treatment response does not necessarily translate into technically and oncologically appropriate resection
Unexpected pathological upstaging	Clinically node-negative disease subsequently found to have pN1/pN2 involvement	Pathological staging may redefine postoperative recurrence risk and treatment indication
Resectability versus operability	Major postoperative morbidity after pneumonectomy following induction treatment	Technical resectability should be distinguished from overall surgical appropriateness and patient operability
Inability to deliver postoperative therapy	Either Comorbidities/frailty or postoperative complications accounted for 25/36 patients not receiving adjuvant therapy	Theoretical eligibility for postoperative treatment does not necessarily translate into real-world treatment feasibility

Abbreviations: *ALK*, anaplastic lymphoma kinase; MDT, multidisciplinary team; and pCR, pathological complete response.

## Data Availability

The data supporting the findings of this study are not publicly available due to patient privacy and confidentiality restrictions. Requests for access may be directed to the corresponding author and will be considered subject to applicable institutional policies and data protection requirements.
